# Supplementary material for: Carbon Quantum Dots from Pomelo Peel as Fluorescence Probes for “Turn-Off–On” High-Sensitivity Detection of Fe3+ and L-Cysteine
Source: Molecules. 2022 Jun 25;27(13):4099. doi: 10.3390/molecules27134099 (PMC9268387; doi:10.3390/molecules27134099)
Supplement: Supplementary file 1 [file molecules-27-04099-s001.zip › molecules-1709329-supplementary.pdf]

Supporting Information

Carbon quantum dots from pomelo peel as fluorescence probes for "turn-off–on" high-sensitivity detection of Fe^{3+} and L-cysteine

Dianwei Zhang, Furui Zhang, Yonghong Liao*, Fenghuan Wang*, and Huilin Liu

Beijing Technology and Business University, 11 Fucheng Road, Beijing, 100048, China.

***Corresponding author: Yonghong Liao**

Tel: (86 10) 68988710

Fax: (86 10) 68985456

Email: liaoyh@th.btbu.edu.cn

***Corresponding author: Fenghuan Wang**

Tel: (86 10) 68985252

Fax: (86 10) 68985456

Email: wangfenghuan@th.btbu.edu.cn

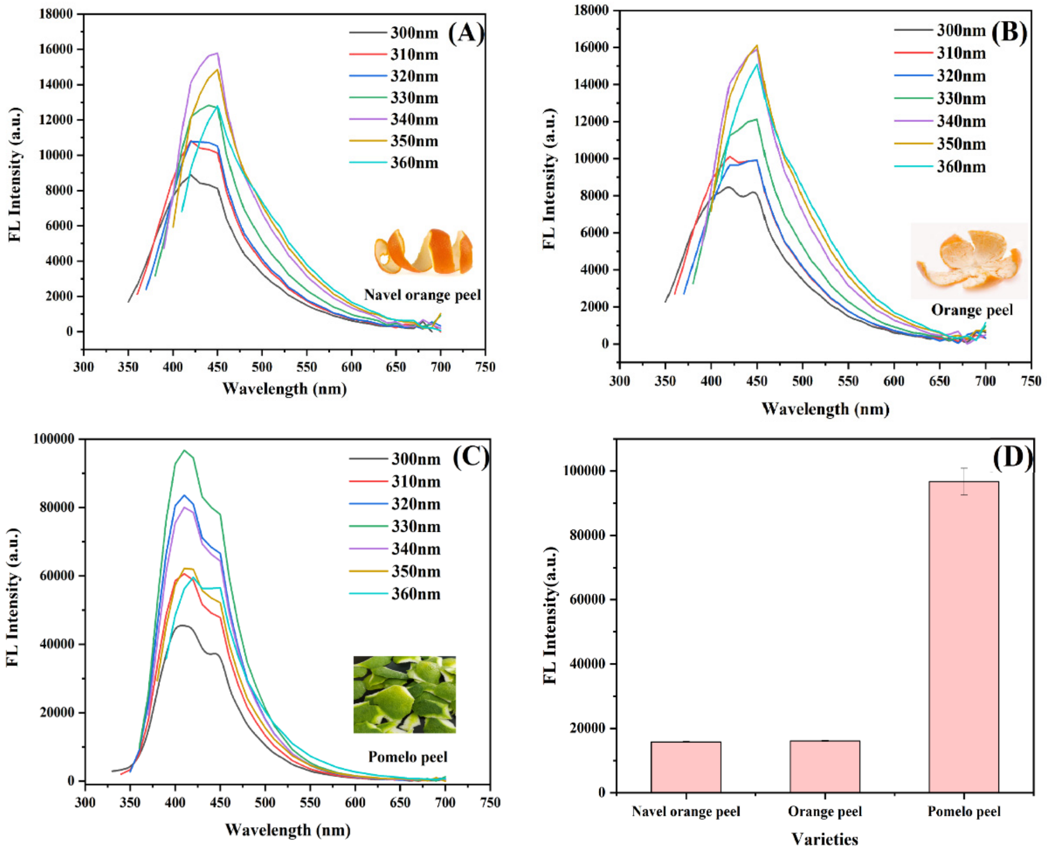

Figure S1 (A) Fluorescence intensity of CQDs from navel orange peel, (B) Fluorescence intensity of CQDs from orange peel, (C) Fluorescence intensity of CQDs from pomelo peel, (D) Fluorescence intensity of CQDs from different carbon sources.

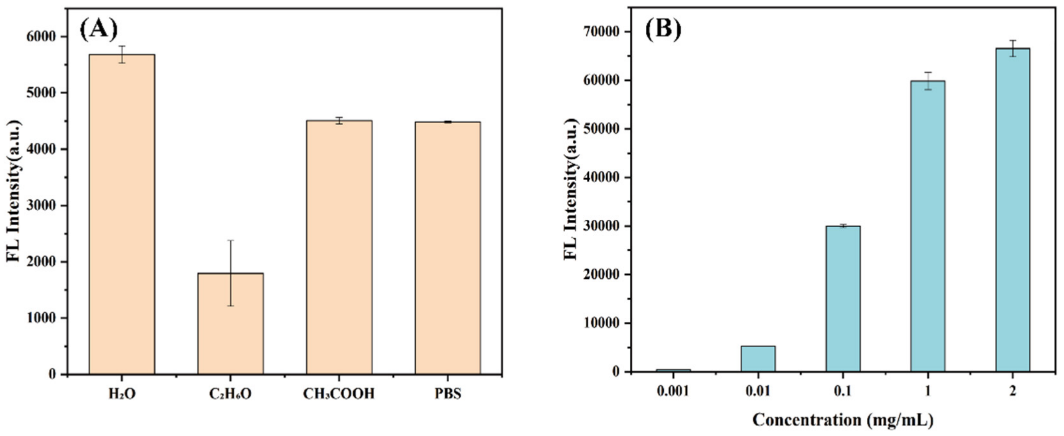

Figure S2 (A) Effect of different solvents on fluorescence intensity of CQDs, (B) Effect of different concentration on fluorescence intensity of CQDs.

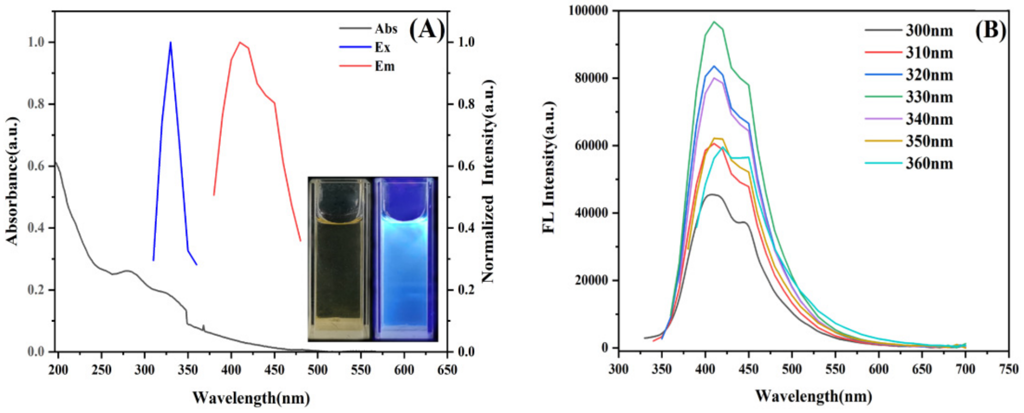

Figure S3 (A) UV absorption spectrum and fluorescence spectra of the optimal excitation and emission of the CQDs. Insert: Photograph of CQDs under the excitation of natural light and UV lamp with 365 nm. (B) Fluorescence emission spectra of the CQDs with a series of excitation wavelengths.

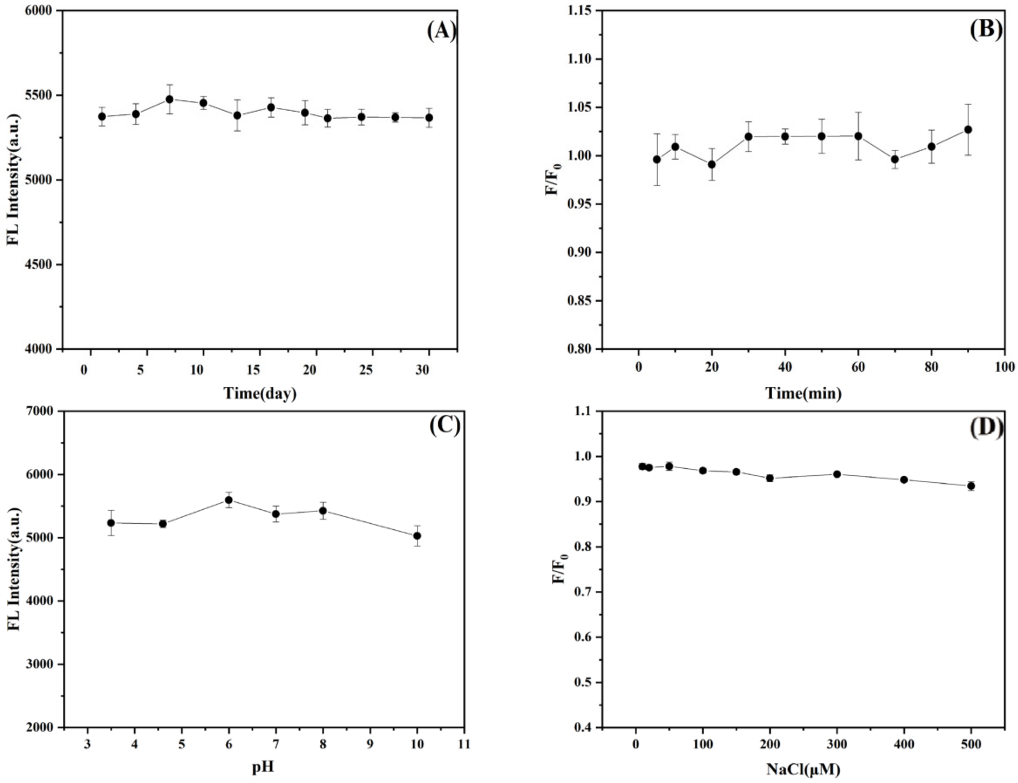

Figure S4 (A) The continuous fluorescence intensity measurements of CQDs aqueous solutions in 30 days. (B) Fluorescence stability of CQDs in aqueous solution with different irradiation time by UV light. (C) Fluorescence intensity of CQDs aqueous solutions at different pH values. (D) Fluorescence stability of CQDs after the addition of different concentrations of NaCl.

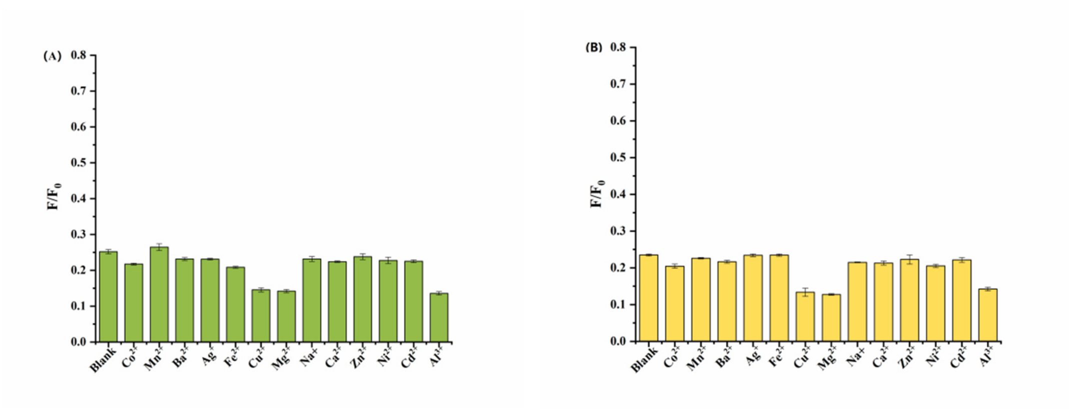

Figure S5 (A) Influences of metal ions with 2 fold concentration of Fe^{3+} on fluorescence intensity of CQDs. (B) Influences of metal ions with 5 fold concentration of Fe^{3+} on fluorescence intensity of CQDs.

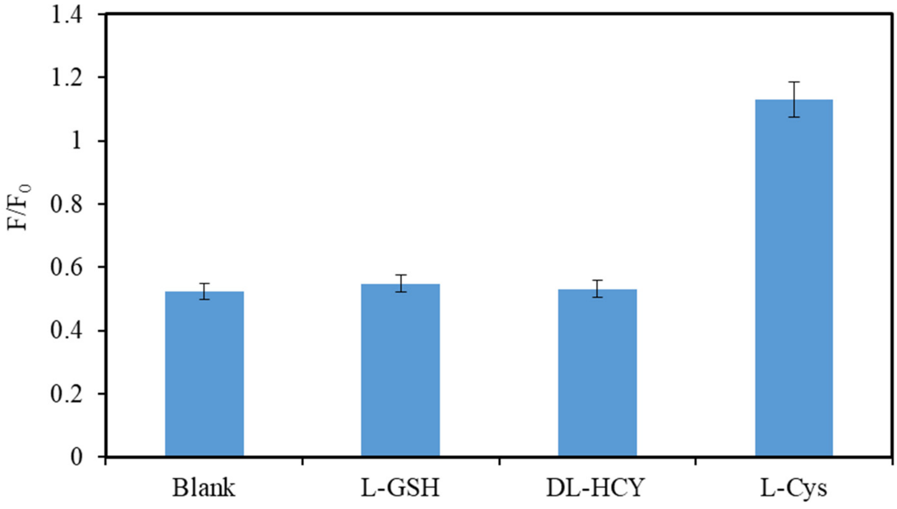

Figure S6 Fluorescence response of the CQDs/Fe^{3+} system in the presence of L-GSH, DL-Hcy and L-Cys.

Table S1 Parameters of fluorescence quantum yield of CQDs.

	Fluorescence integral area	Absorbance	QY
Quinine Sulfate	118747547.5	0.1	56%
CQDs	23127302.5	0.063	17.31%

Table S2 Recoveries of Fe^{3+} and L-Cys from spiked water and amino acid beverage samples.

Samples	Targets	Added (μM)	Detection (μM)	Recovery (%)	RSD (%)
Water	Fe^{3+}	10	8.35	83.47	2.156
		30	31.95	106.53	3.193
		60	58.12	96.87	0.685
Amino acid beverage	L-Cys	10	12.27	122.74	3.981
		30	27.07	90.22	0.358
		60	52.25	87.08	1.106

Table S3 The repeatability of the CQDs-Fe^{3+} system for L-Cys detection.

n	Fluorescence intensity	RSD (%)
1	2038	
2	2041	
3	2048	0.23
4	2035	
5	2042	
6	2045	
